# Incorporating Issues of Elderly Loneliness into the Coronavirus Disease–2019 Public Health Response

**DOI:** 10.1017/dmp.2020.145

**Published:** 2020-05-07

**Authors:** Sonny S. Patel, Aaron Clark-Ginsberg

**Affiliations:** Department of Global Health and Population, Harvard T.H. Chan School of Public Health, Boston, MA; Harvard Humanitarian Initiative, Harvard University, Cambridge, MA; RAND Corporation, Santa Monica, CA

**Keywords:** COVID-19, decision-making, elderly, loneliness, public health emergency response, resilience, vulnerable populations

## Abstract

As the systems that people depend on are increasingly strained by the coronavirus disease–2019 (COVID-19) outbreak, public health impacts are manifesting in different ways beyond morbidity and mortality for elderly populations. Loneliness is already a chief public health concern that is being made worse by COVID-19. Agencies should recognize the prevalence of loneliness among elderly populations and the impacts that their interventions have on loneliness. This letter describes several ways that loneliness can be addressed to build resilience for elderly populations as part of the public health response to COVID-19.

As the systems that people depend on are increasingly strained by the coronavirus disease–2019 (COVID-19) outbreak, public health impacts are manifesting in different ways beyond morbidity and mortality. For elderly populations (people age 60 years and older), loneliness is already a chief public health concern that is being made worse by COVID-19. To be sure, morbidity and mortality must be addressed as part of the public health response to COVID-19; elderly mortality rates range from 3.6% to 14.8% in China and are similar in other countries. Yet to protect the elderly, public health agencies must also address how COVID-19 interventions designed to reduce mortality can contribute to loneliness. How can we plan a better response for the elderly population where we do not further harm with infection or increase loneliness?

Loneliness, the negative feelings associated with perceived social isolation, is already a severe public health concern for elderly populations.^1^ Loneliness is associated with reduced happiness and satisfaction with life, and depression, which can manifest in physical health problems.^2^ Because of this, many social service programs for the elderly have programs centered around in-person social interactions. By reducing in-person social interactions in the name of physical distancing, the COVID-19 crisis is expected to increase loneliness among the elderly.^3^

There are several ways that loneliness can be addressed as part of the public health response to COVID-19. First, public health agencies should work to identify how interventions designed to lessen the spread of COVID-19, such as physical distancing, might contribute to loneliness, and work mitigate those effects. Second, the agencies can change how they deliver support to elderly. Research on other disasters in other contexts shows that implementing interventions compassionately and with the appropriate cultural competencies can go far in ensuring that emotional and physical needs are met during times of crisis.^4,5^ Third, they can implement programs to intervene directly to reduce loneliness among elderly, and do so in ways that minimize chances for COVID-19 spread. For instance, elderly can be provided with and trained in the use of technologies like online conferencing systems to combat loneliness via remote interventions. While this suite of interventions could go far in addressing loneliness, reducing COVID-19 spread might still require interventions like physical distancing that could increase loneliness. Agencies should recognize these impacts and include them as part of their cost-benefit equations for response decisions.
